# Reproductive factors and oesophageal cancer in Chinese women: a case-control study

**DOI:** 10.1186/1471-230X-11-49

**Published:** 2011-05-09

**Authors:** Zu-Hui Chen, Jian-Li Shao, Jin-Rong Lin, Xia Zhang, Qing Chen

**Affiliations:** 1 College of Clinical Medicine, Jinan University, No. 613, Huangpu Avenue, 510630 Guangzhou, China; 2 School of Public Health and Tropical Medicine, Southern Medical University, No. 1838, Guangzhou Avenue, 510515 Guangzhou, China

## Abstract

**Background**

Previous studies showed that sex hormone might play a role in the development of oesophageal cancer in Western countries. However, evidence from Chinese populations is still lacking.

**Methods**

We performed a hospital-based case-control study in Guangzhou, China. From June 2006 to May 2009, face-to-face interviews were conducted on 73 cases and 157 controls. Cases were Chinese females with newly diagnosed primary oesophageal cancer. Controls were hospitalized individuals without cancer and frequency matched by age groups. The interviews included questions about childbearing and menarche history, together with potential confounders. Unconditional logistic regression was used to estimate the risk of factors.

**Results**

Women who had given birth before were not at increased risk compared to childless women (adjusted OR = 1.17, 95% CI: 0.48 ~ 2.85). The risk of oesophageal cancer increased with age at first birth: the adjusted OR for women first giving birth at age 25 or later was 2.02 (95% CI: 1.01 ~ 4.04) compared with those reporting their first birth before age 22. History of spontaneous abortion was not significantly associated with increased risk (adjusted OR = 1.37, 95% CI: 0.49 ~ 3.83). No significant association was observed between menstrual variables (age at menarche, age at menopause, and years of menstruation) and risk of oesophageal cancer.

**Conclusions**

Giving birth at later age may increase the risk of oesophageal cancer in women. Further studies in Chinese populations with larger sample sizes are still needed.

## Background

Worldwide, oesophageal cancer is the eighth most common type of cancer and the sixth most common cause of death from cancer [1]. In China overall, oesophageal cancer ranks second in incidence and it is estimated that almost half of all oesophageal cancer cases in the world are contributed by China [2]. Oesophageal cancer remains an important public health problem in China.

Oesophageal cancer occurs mainly in two histological types, squamous cell carcinoma and adenocarcinoma. Squamous cell carcinoma has always been the dominant histological type of oesophageal cancer in China. However, the incidence of adenocarcinoma has increased rapidly in some developed countries during the past decades. Meanwhile, the incidence of squamous cell carcinoma has been more stable over time in these countries [3]. Such inconsistent secular trends of different histological types of oesophageal carcer in these countries suggested the differences in etiology by histological type, which were also supported by many analytical epidemiological studies [4-6].

One of the most intriguing observations in the incidence of oesophageal cancer is the male predominance, which has not been well explained. In Western countries, the incidence of oesophageal cancer has a male to female ratio of up to 8:1 [7,8]. This strong male predominance in Western countries is also age-related [9,10], differs by histological type [7], but seems unexplained by the differences in the prevalence of some known risk factors between males and females [5]. The male to female ratio of oesophageal cancer incidence rates is much lower in Asian countries. In 2008, the male to female ratio of oesophageal cancer incidence rates was about 2 in China [1]. It is possible that differences in extrinsic exposures, such as cigarette smoking and alcohol drinking, may explain part of the difference in esophageal cancer incidence rates in men and women in China. It was also hypothesized that some intrinsic exposures, such as sex hormone may play a role in the development of oesophageal cancer. This hypothesis has been tested further in Western countries. In a case-control study in England and Scotland [11], breastfeeding was associated with a 60% reduction in oesophageal adenocarcinoma risk among women. A pooled analysis based on various case-control studies conducted in Italy found a positive trend toward an increase risk of oesophageal cancer with increasing parity [12]. However, in a Swedish case-control study, no association between childbearing and oesophageal adenocarcinoma was found [13]. A recent pooled analysis also suggested that the endogenous reproductive factors such as parity, menstruation history of pregnancy were not statistically significantly associated with oesophageal adenocarcinoma [14]. Findings of previous studies remain inconsistent.

Although incidence of oesophageal cancer in China is among the highest in the world, no evidence is available on the effect of sex hormone on oesophageal cancer risk among Chinese populations. In addition, squamous cell oesophageal cancer represents the majority of oesophageal cancer cases in China. Thus, previous evidences from Western countries mostly concerning the effect of reproductive factors on the risk of oesophageal adenocarcinoma may not apply to Chinese populations. Therefore, we conducted a case-control study in Guangzhou, China, to investigate the long-term effects of reproductive factors on oesophageal cancer risk in women in a Chinese population.

## Methods

### Study population

This study was conducted in one large teaching hospital in Guangzhou, China. Guangzhou is a city with moderate incidence rates for oesophageal cancer in China. In 2006, there were totally 257 male and 56 female cases diagnosed with oesophageal cancer in Guangzhou with crude incidence rates of 13.04/100.000 and 2.97/100,000, respectively, showing a male/female incidence ratio of 4.4 [15]. All Chinese female cases with newly diagnosed primary oesophageal cancer between June 2004 and May 2010 in the hospital were invited for face-to-face interviews within two months after diagnosis. All cases recruited in this study were examined endoscopically and histologically confirmed. Among a total of 81 eligible cases, 73 were interviewed with a participation rate of 90%. Controls were randomly selected from people who requested general health examinations in the same hospital during the same period. Controls were required to be without any history of any type of cancer and frequency matched by five-year age groups, with a control-to-case ratio of two, whenever possible. Among a total of 178 eligible controls, 157 were successfully interviewed with a participation rate of 88%.

### Data collection

Trained interviewers conducted face-to-face interviews using a structured questionnaire to collect information on childbearing and menarche history, together with sociodemographic characteristics and potential confounding factors. Collected potential confounders mainly included tobacco smoking, alcohol use, gastroesophageal reflux, anthropometric measures (height and weight), dietary habits, and regular medication. Similar to previous studies [4,13], history of gastroesophageal reflux was estimated by asking subjects about recurrent heartburn and regurgitation, which are the cardinal symptoms of gastroesophageal reflux, occurring at least once weekly at least 5 years before the diagnosis (for cases) or the interview (for controls). To avoid reverse causality, subjects were asked to report their anthropometric measures five years before the diagnosis (for cases) or the interview (for controls). Approval to conduct this study was granted by the Ethics Committee of Southern Medical University (Code No. EC2005.097) and the Ethics Committee for Clinical Research of the First Affiliated Hospital of Jinan University (Code No. CRE 2006.019). I Informed consent was obtained before each interview.

### Statistical analysis

All statistical analyses were performed using the Statistical Package for the Social Sciences (SPSS) software 13.0 for windows. Chi square or t tests were used to test differences of sociodemographic factors and potential confounders between the cases and controls. Unconditional logistic regression was used to estimate the odds ratios (ORs) and corresponding 95% confidence intervals (CIs) for each exposure variable. The associations between exposure variables and risk of oesophageal cancer were further examined after adjusting for potential confounders using multivariate logistic regression models. Potential confounders entered into the final model included: age, reflux, smoking, alcohol use, education, employment, body mass index, and intake of fresh vegetables and fruits. We also considered further adjustment for aspirin or nonsteroidal anti-inflammatory drugs (NSAID) use but the estimated ORs did not substantially changed. Also because only a very small proportion of the subjects (lower than 10% for both cases and controls) had regular use of such medicines, aspirin or NSAID use was not included in the final models. The cut-off points for number of children born and age at menarche were predefined based on biological considerations. Age at first birth, age at menopause, and years of menstruation were grouped into quartiles using cut-off points among the controls. To see whether the risk estimates differ by histological type, we also re-analyzed the data after restricting cases to those with diagnosis of squamous cell carcinoma.

## Results

### Subject characteristics

Squamous cell carcinoma was the most frequent histological type, which comprised 93.2% (68 cases) of all cases. The distribution of demographic characteristics and major risk factors for oesophageal cancer among cases and controls are shown in Table 1. The mean age was 63.4 years among cases and 62.7 years among controls. There were no significant differences between cases and controls in smoking status, body mass index, intake of fresh vegetables and fruits. More cases than controls had never been employed (P = 0.047) and were less educated (P = 0.038). Reflux was also more common among cases than controls (P < 0.001).

**Table 1:** Selected characteristics of cases and controls

Variable	Cases (N = 73)	Controls (N = 157)	P value
Age, years, mean ± SD	63.4 ± 10.3	62.7 ± 11.4	0.656
Education less than middle school, n (%) *	19 (26.0)	23 (14.6)	0.038
Never employed, n (%) *	15 (20.5)	17 (10.8)	0.047
Smoking, n (%)	6 (8.2)	8 (5.1)	0.356
High alcohol intake, n (%)	5 (6.8)	9 (5.7)	0.742
Reflux, n (%) *	29 (39.7)	16 (10.2)	<0.001
Overweight or obesity, n (%)	28 (38.4)	63 (40.1)	0.798
Low consumption of fresh vegetables, n (%)	6 (8.2)	10 (6.4)	0.891
Low consumption of fresh fruits, n (%)	10 (13.7)	17 (10.8)	0.529

SD: Standard Deviation. * P < 0.05.

Smoking was defined as having ever smoked over 100 cigarettes or equivalent. High alcohol intake was defined as consumption of at least 70g pure alcohol per week. Reflux referred to symptoms occurring at least 5 years before interview. Overweight or obesity meant body mass index >25 kg/m^2^. Low consumption of fresh vegetables or fruits was that less than weekly.

### Reproductive factors and risk of oesophageal cancer

The relationship between oesophageal cancer risk and reproductive factors is presented in Table 2. Women who had given birth before were not at increased risk compared to childless women (adjusted OR = 1.17, 95% CI: 0.48 ~ 2.85). No significant association was found between number of children born and risk of oesophageal cancer. The risk of oesophageal cancer increased with age at first birth: the adjusted OR for women first giving birth at age 25 or later was 2.02 (95% CI: 1.01 ~ 4.04) compared with those reporting their first birth before age 22. History of spontaneous abortion was not significantly associated with increased risk (adjusted OR = 1.37, 95% CI: 0.49 ~ 3.83). No significant association was observed between menstrual variables (age at menarche, age at menopause, and years of menstruation) and risk of oesophageal cancer. Results of sensitivity analysis restricted to the dominant histological type, squamous cell carcinoma, revealed no strong difference from the pooled results of all histological types.

**Table 2:** Reproductive factors and risk of oesophageal cancer among female participants

Variable	Controls n (%)	All types of oesophageal cancer			Squamous cell oesophageal cancer		
		
		Cases n (%)	Crude OR (95% CI)	Adjusted OR* (95% CI)	Cases n (%)	Crude OR (95% CI)	Adjusted OR* (95% CI)
Children							
No	23	9	1	1	8	1	1
Yes	134	64	1.22 (0.53, 2.78)	1.17 (0.48, 2.85)	60	1.29 (0.54, 3.04)	1.20 (0.47, 3.36)
Number of children born							
0	23	9	1	1	8	1	1
1 or 2	88	45	1.26 (0.71, 2.22)	1.31 (0.72, 2.35)	43	1.40 (0.58, 3.39)	1.34 (0.67, 2.68)
3 or more	46	19	1.02 (0.51, 2.03)	1.03 (0.49, 2.14)	17	1.06 (0.40, 2.82)	1.02 (0.38, 2.74)
Age at first birth							
<22 years	51	17	1	1	15	1	1
22-24 years	46	20	1.30 (0.61, 2.78)	1.27 (0.54, 2.98)	19	1.40 (0.64, 3.08)	1.31 (0.50, 3.43)
>24 years	37	27	2.19 (1.05, 4.58)	2.02 (1.01, 4.04)	26	2.39 (1.11, 5.12)	2.12 (1.02, 4.41)
Spontaneous abortion							
No	129	55	1	1	51	1	1
Yes	28	18	1.51 (0.77, 2.94)	1.37 (0.49, 3.83)	17	1.54 (0.77, 3.04)	1.39 (0.50, 3.86)
Age at menarche							
<12 years	19	11	1	1	10	1	1
12-16 years	128	47	0.63 (0.28, 1.43)	0.81 (0.38, 1.86)	44	0.65 (0.28, 1.51)	0.80 (0.37, 1.72)
>16 years	10	15	2.59 (0.87, 7.71)	2.15 (0.77, 6.00)	14	2.66 (0.87, 8.11)	2.19 (0.79, 6.07)
Age at menopause							
<46 years	44	19	1	1	18	1	1
46-48 years	55	31	1.31 (0.65, 2.61)	1.12 (0.40, 3.14)	29	1.29 (0.63, 2.61)	1.10 (0.35, 2.86)
>48 years	58	23	0.92 (0.45, 1.89)	0.96 (0.33, 2.79)	21	0.89 (0.42, 1.85)	0.94 (0.31, 2.85)
Years of menstruation							
<32 years	41	20	1	1	19	1	1
33-36 years	60	28	0.96 (0.48, 1.92)	0.94 (0.41, 2.15)	25	0.90 (0.44, 1.84)	0.91 (0.39, 2.12)
>36 years	56	25	0.92 (0.45, 1.86)	0.93 (0.38, 2.27)	24	0.92 (0.45, 1.90)	0.92 (0.35, 2.42)

* Adjusted for age, reflux, smoking, alcohol use, education, employment, body mass index, and intake of fresh vegetables and fruits.

## Discussion

The present study was the first one that has been performed in a Chinese population to investigate the effects of reproductive factors on oesophageal cancer risk. This study suggested that giving birth at later age might increase the risk of oesophageal cancer in women. Such findings were in line with those of a previous study in Italy that the OR was 2.5 for women first giving birth at age 30 or later compared with those whose first birth occurred before age 25 [12]. We did not find significant association of oesophageal cancer risk with parity or menarche history. However, a previous analysis based on three case-control studies in Italy and Switzerland showed that the OR was higher for parous than for nulliparous women and age at menopause was inversely associated with squamous cell oesophageal cancer risk [16]. The inconsistency of these studies may be explained by differences in population background, study design, sample size, and also by chance. Further confirmation of existing findings is still needed in future studies.

This study has several major strengths. First, an extensive effort was made to collect information on major risk factors for oesophageal cancer, which was further considered and adjusted throughout the analysis. Second, all cases in this study were histologically confirmed, which minimized misclassification. In addition, in many hospital-based studies, controls were taken from hospitalized individuals with higher chance than the general population to share a common exposure with cases, which may be a threat to the validity of the results. In this study controls were selected from those who came to hospitals for routine health examination, probably making the controls more representative of the general population. However, since it was not a random sample of the general population, there was still a certain risk of selection bias if people seeking routine health examination had any difference in terms of the studied exposures.

Because of the rarity of oesophageal cancer in women, we only had limited number of cases. Actually, other similar studies also suffered from this problem with low statistical power [13]. Increasing the number of controls to a control-case ratio of 4 can, at least to some extent, increase the study power, which needs consideration in future studies. Furthermore, existing evidences showed that the two histological types of oesophageal cancer, squamous cell carcinoma and adenocarcinoma, differ substantially in etiology [4-6]. However, sensitivity analysis restricted to the dominant histological type, squamous cell carcinoma, revealed no strong difference, but the statistical power was even lower. Obviously, studies with larger sample size are still needed.

## Conclusions

As the first study to investigate the effects of reproductive factors on oesophageal cancer risk in a Chinese population, this study found suggestive evidence that giving birth at later age might increase the risk of oesophageal cancer in women though this needed confirmation. Further studies in Chinese populations with larger sample sizes are still warranted.

## Competing interests

The authors declare that they have no competing interests.

## Authors' contributions

All authors read and approved the final study. ZHC designed and coordinated the study, carried out data analysis and wrote the draft. JLS, JRL, and XZ recruited subjects, organized interviews, and participated in data analysis. QC participated in study design and coordination, and edited the manuscript.

## Pre-publication history

The pre-publication history for this paper can be accessed here:

http://www.biomedcentral.com/1471-230X/11/49/prepub
